# KAT8 drives M2 macrophage polarization to exacerbate allergic airway inflammation

**DOI:** 10.1016/j.isci.2026.115348

**Published:** 2026-03-12

**Authors:** Xianwen Lai, Han Li, Zhao Zhao, Yu Zhong, Guomei Su, Jiewen Huang, Yuanyuan Xiang, Ruina Huang, Jingyun Quan, Zhihang Feng, Zhenfu Fang, Shihai Li, Tong Huang, Zhiling Xiong, Yuting Lei, Wenchao Zhang, Jielin Duan, Xiao Gao, Tianwen Lai

**Affiliations:** 1 Dongguan Key Laboratory of Immune Inflammation and Metabolism, The First Dongguan Affiliated Hospital, Guangdong Medical University, Dongguan 523121, China; 2 Department of Respiratory and Critical Care Medicine, The First Dongguan Affiliated Hospital, Guangdong Medical University, Dongguan 523121, China; 3 School of Ocean and Tropical Medicine. Guangdong Medical University, Zhanjiang, Guangdong 524023, China; 4 Clinical Research Center, Affiliated Hospital of Guangdong Medical University, Zhanjiang 524001, China; 5 School of Basic Medicine, Guangdong Medical University, Dongguan 523808, China; 6 Department of Geriatrics, Affiliated Hospital of Guangdong Medical University, Zhanjiang 524001, China; 7 Institute of Respiratory Diseases, The Affiliated Hospital of Guangdong Medical University, Zhanjiang 524001, China

**Keywords:** immunology, respiratory medicine, biological sciences

## Abstract

Dysregulated macrophage polarization is a pivotal driver of airway inflammation in asthma, yet the underlying molecular mechanisms remain incompletely understood. Here, we demonstrate that histone acetyltransferase KAT8 exacerbates allergic airway inflammation by promoting M2 macrophage polarization in asthma. KAT8 expression was significantly upregulated in lung macrophages of asthmatic mice and in bone marrow-derived macrophages (BMDMs) stimulated with house dust mite (HDM). Macrophage-specific KAT8 deficiency attenuated allergic airway inflammation and inhibited M2 macrophage polarization by suppressing signal transducer and activator of transcription 3 (STAT3) signaling. Mechanistically, KAT8 directly interacted with STAT3 and targeted it for acetylation, thereby driving M2 macrophage polarization. Importantly, pharmacological inhibition of KAT8 reduced M2 macrophage polarization and attenuated allergic airway inflammation. These findings establish KAT8 as a critical regulator of macrophage-driven allergic inflammation via STAT3 acetylation, highlighting its potential as a therapeutic target for asthma.

## Highlights

• KAT8 drives allergic airway inflammation by promoting M2 macrophage polarization
• KAT8 interacts with and acetylates STAT3, enhancing the M2 macrophage program
• Genetic and pharmacological KAT8 inhibition alleviates allergic airway inflammation

## Graphical abstract

**Figure:**
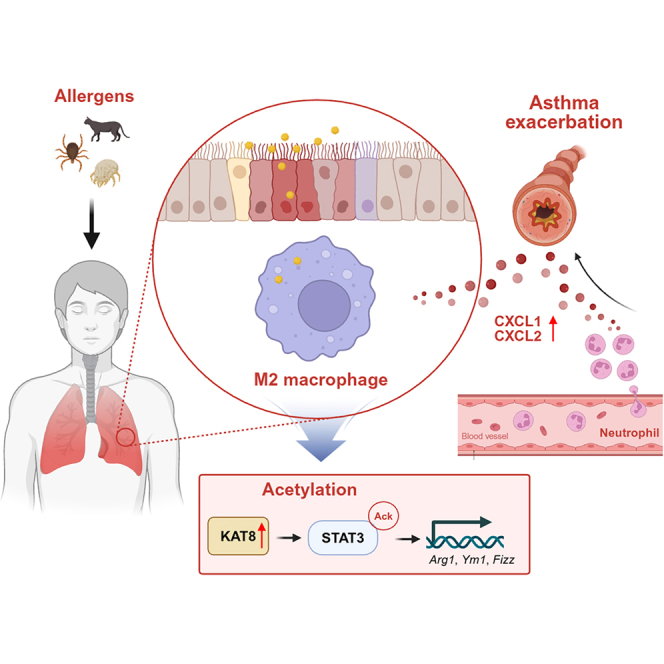

## Introduction

Asthma is a heterogeneous chronic lung disease affecting over 300 million people worldwide, clinically manifested by wheezing, coughing, shortness of breath, and chest tightness.^1^ In addition to the well-established eosinophilic phenotype, neutrophilic infiltration represents another key characteristic, particularly in severe asthma, leading to goblet cell hyperplasia and mucus production, airway remodeling, and hyperresponsiveness.^2^^,^^3^ Despite substantial advances in understanding asthma pathogenesis, the molecular mechanisms regulating airway neutrophil infiltration in asthma remain incompletely understood.

Macrophages, central orchestrators of asthma pathogenesis, can regulate allergic airway inflammation by releasing various cytokines and chemokines or presenting allergens.^4^ Macrophages exhibit remarkable plasticity and can be polarized into pro-inflammatory M1 or immunoregulatory M2 subtypes in response to different microenvironmental cues.^5^^,^^6^ In asthma, allergen exposure drives IL-4/IL-13-dependent M2 polarization, amplifying allergic airway inflammation and promoting asthma progression.^7^^,^^8^ Thus, elevated M2 macrophage frequencies are observed in patients with asthma, which correlate with disease severity and poor clinical outcomes.^9^^,^^10^^,^^11^ Research, including our own, has highlighted the essential role of M2 macrophages in asthma pathogenesis, contributing to hyperresponsiveness, airway inflammation, and remodeling.^9^^,^^12^ Thus, the inhibition of M2 macrophage polarization can alleviate allergen-mediated airway inflammation in asthma mouse models. However, the molecular mechanism regulating M2 macrophage polarization remains incompletely defined.

Emerging evidence highlights dysregulated protein acetylation as a hallmark of asthma.^13^ Acetylation dynamics, controlled by histone acetyltransferases (HATs) and deacetylases (HDACs), regulate chromatin accessibility, transcription factor activity, and metabolic reprogramming.^14^ KAT8, a lysine acetyltransferase in the MYST family, can acetylate both histones and non-histone substrates, mediating diverse biological processes, such as embryogenesis, oncogenesis, and immune regulation.^15^^,^^16^^,^^17^ Recently, *Liu* et al. have reported that KAT8 can induce IL-33 acetylation in airway epithelial cells, which is essential for IL-33 cleavage and release, and inhibiting KAT8-mediated IL-33 acetylation could alleviate allergic airway inflammation and airway hyperresponsiveness,^18^ indicating the involvement of KAT8 in asthma development. However, whether KAT8 regulates the function of other cell types, such as macrophages, to participate in asthma pathogenesis has not yet been reported.

Here, we identified KAT8 as a critical driver of M2 macrophage polarization and airway neutrophilic inflammation using a house dust mite (HDM)/lipopolysaccharide (LPS) induced neutrophil-dominant asthma model. Allergen exposure upregulated KAT8 expression in macrophages both *in vivo* and *in vitro*. Macrophage-specific KAT8 deficiency inhibited M2 macrophage polarization and mitigated allergic airway inflammation. KAT8 interacted with signal transducer and activator of transcription 3 (STAT3) and targeted it for acetylation. Critically, the pharmacological inhibition of KAT8 reduced airway inflammation *in vivo*, highlighting its potential as a therapeutic target for asthma.

## Results

### Allergen exposure induces KAT8 expression in macrophages

**Figure 1:**
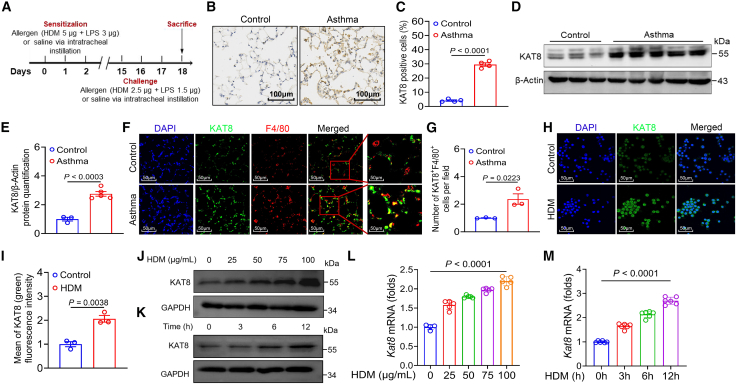
Allergen exposure induces KAT8 expression in macrophages (A) Schematic view of the asthma murine model induced by the intratracheal instillation of HDM and LPS. (B–E) KAT8 expression in lung tissues (n = 3–5), assessed by IHC (B, C; ×200 magnification) and WB (D, E). Scale bars, 100 μm. (F and G) IF staining analyzes co-localization of KAT8 (green) with macrophages (F4/80, red) in lung tissues (*n* = 3), and quantified in (G). Scale bars, 50 μm. (H and I) KAT8 expression (green) in HDM-stimulated MH-S cells, assessed by IF staining. Scale bars, 50 μm. (J–M) Dose- and time-dependent upregulation of KAT8 expression in HDM-stimulated BMDMs, analyzed by WB (J, K) and qRT-PCR (L, M). Data are presented as mean ± SEM, representing two or three independent experiments (Student’s t tests and ANOVA tests).

To investigate the role of KAT8 in asthma, we established a neutrophil-dominant murine asthma model via co-exposing to HDM and LPS as previously described (Figure 1A).^9^^,^^19^ We determined KAT8 expression in mouse lung tissues by immunohistochemistry (IHC), and observed a significant upregulated KAT8 expression in the lung from asthmatic mice compared to that in control mice (Figures 1B and 1C). Western blot (WB) result confirmed the increased KAT8 expression in the lung from asthmatic mice (Figures 1D and 1E).

To identify which immune cell populations are responsible for the elevated KAT8 expression, we performed double immunofluorescence (IF) staining of a lung section. We observed the upregulated KAT8 expression (green) in macrophages (F4/80, red) and other cell types (F4/80^-^) in lung tissues of asthmatic mice (Figures 1F and 1G). In line with *in vivo* observations, IF staining confirmed the increased KAT8 expression in the murine alveolar macrophage cell line (MH-S cells) after HDM stimulation (Figures 1H and 1I). Further, HDM stimulation induced KAT8 expression in a time- and dose-dependent manner in BMDMs (Figures 1J–1M) and MH-S cells (Figures S1A–S1D). Taken together, these findings indicate that allergen exposure induces KAT8 expression in macrophages both *in vivo* and *in vitro*.

### KAT8 deficiency in macrophages attenuates allergic airway inflammation

**Figure 2:**
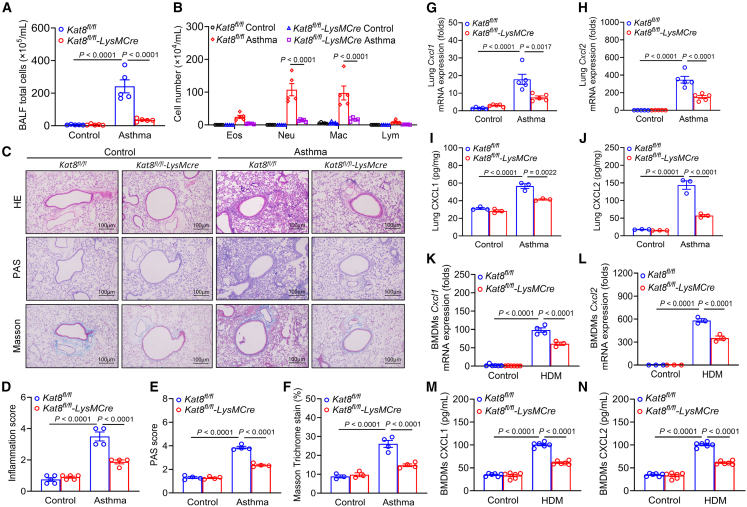
KAT8 deficiency in macrophages attenuates allergic airway inflammation (A and B) Total cell counts (A) and differential cell counts (B) in BALF from *Kat8*^*fl/fl*^ and *Kat8*^*fl/fl*^*-LysMCre* asthmatic mice (*n* = 5). (C–F) Airway inflammation, collagen deposition, and mucus production in lung tissues from *Kat8*^*fl/fl*^ and *Kat8*^*fl/fl*^*-LysMCre* asthmatic mice (*n* = 4), assessed by HE (C, D), Masson’s trichrome (C, E), and periodic acid-Schiff (C, F) staining. Scale bars, 100 μm. (G–J) CXCL1 and CXCL2 expression and production in lung tissues from *Kat8*^*fl/fl*^ and *Kat8*^*fl/fl*^*-LysMCre* asthmatic mice (n = 3–5), assessed by qRT-PCR (G, H) and ELISA (I, J). (K–N) CXCL1 and CXCL2 expression and production in HDM-stimulated BMDMs, analyzed by qRT-PCR (K, L) and ELISA (M, N). Data are presented as mean ± SEM, representing two or three independent experiments (ANOVA tests).

To assess the role of KAT8 in asthma *in vivo*, we crossed *Kat8*^*fl/fl*^ mice with LysMCre mice to generate macrophage-specific *Kat8* knockout mice (*Kat8*^*fl/fl*^*-LysMCre*), which were confirmed by genotyping, WB, and qRT-PCR (Figures S2A–S2D). *Kat8*^*fl/fl*^*-LysMCre* asthmatic mice showed significantly fewer total cell count, neutrophils, and macrophages in bronchoalveolar lavage fluid (BALF) compared to *Kat8*^*fl/fl*^ asthmatic mice (Figures 2A and 2B). Histopathological analyses revealed that *Kat8*^*fl/fl*^*-LysMCre* asthmatic mice showed a marked reduction in airway inflammation, mucus secretion, and collagen deposition in the lung compared with those observed in *Kat8*^*fl/fl*^ asthmatic mice (Figures 2C–2F). Consistent with this attenuated phenotype, the expression and production of CXCL1 and CXCL2 were dramatically reduced in the lung of *Kat8*^*fl/fl*^*-LysMCre* asthmatic mice compared to the asthmatic littermates (Figures 2G–2J). However, the expression of *Tnf-α*, *Il-1β,* and *Il-6* in lung tissues was comparable between *Kat8*^*fl/fl*^ and *Kat8*^*fl/fl*^*-LysMCre* asthmatic mice (Figures S2E–S2G). Consistent with *in vivo* data, KAT8 deficiency profoundly decreased CXCL1 and CXCL2 expression and production in BMDMs after HDM stimulation (Figures 2K–2N), without significant effects on *Tnf-α*, *Il-1β,* and *Il-6* expression (Figures S2H–S2J). Moreover, the expression and production of CXCL1 and CXCL2 had no statistical difference in neutrophils isolated from *Kat8*^*fl/fl*^ and *Kat8*^*fl/fl*^*-LysMCre* mice (Figures S2K–S2N). These data suggest that KAT8 deficiency in macrophages alleviates allergen-induced airway inflammation.

### KAT8 promotes allergen-induced M2 macrophage polarization

**Figure 3:**
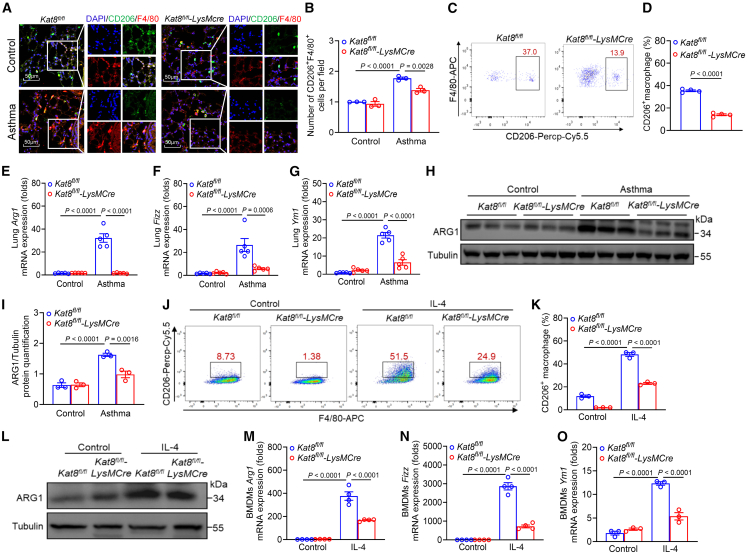
KAT8 promotes allergen-induced M2 macrophage polarization (A and B) M2 macrophages (CD206^+^F4/80^+^) in the lung of *Kat8*^*fl/fl*^ and *Kat8*^*fl/fl*^*-LysMCre* asthmatic mice (*n* = 3), assessed by IF staining. Scale bars, 50 μm. (C and D) Flow cytometry analysis of CD206^+^ macrophages in lung tissues of *Kat8*^*fl/fl*^ and *Kat8*^*fl/fl*^*-LysMCre* asthmatic mice (*n* = 4), and quantified in (D). (E–I) M2 hallmark genes (*Arg1*, *Fizz1*, *Ym1*) expression (E-G) and ARG1 protein levels (H, I) in lung tissues from *Kat8*^*fl/fl*^ and *Kat8*^*fl/fl*^*-LysMCre* asthmatic mice (n = 3–5). (J and K) Flow cytometry analysis of CD206^+^ macrophages in IL-4-stimulated BMDMs isolated from *Kat8*^*fl/fl*^ and *Kat8*^*fl/fl*^*-LysMCre* mice (*n* = 3), and quantified in (K). (L–O) ARG1 protein levels (L) and M2 hallmark genes (*Arg1*, *Fizz1*, *Ym1*) expression (M-O) in IL-4-stimulated BMDMs isolated from *Kat8*^*fl/fl*^ and *Kat8*^*fl/fl*^*-LysMCre* mice. Data are presented as mean ± SEM and represent two or three independent experiments (Student’s t tests and ANOVA tests).

Previous studies have shown that HDM exposure drives M2 polarization, a critical driver for allergic airway inflammation.^12^ To investigate whether KAT8 influences macrophages polarization, we performed IF staining to analyze M1 (iNOS^+^F4/80^+^) and M2 (CD206^+^F4/80^+^) macrophages in lung tissues of *Kat8*^*fl/fl*^ and *Kat8*^*fl/fl*^*-LysMCre* asthmatic mice. IF staining revealed a marked reduction in M2, but not M1 macrophage accumulation in the lung of *Kat8*^*fl/fl*^*-LysMCre* asthmatic mice (Figures 3A, 3B, S3A, and S3B). Flow cytometry further confirmed the decreased frequency of M2 macrophages (CD206^+^ macrophages) in lung tissues of *Kat8*^*fl/fl*^-*LysMCre* asthmatic mice (Figures 3C, 3D, and S3C). Consistently, the mRNA abundance of classical M2 hallmark genes (*Arg1*, *Fizz1*, and *Ym1*) and ARG1 protein levels, but not *Nos2* (M1 marker gene), were downregulated in the lung of *Kat8*^*fl/fl*^-*LysMCre* asthmatic mice (Figures 3E–3I and S3D). To validate these *in vivo* findings, BMDMs from *Kat8*^*fl/fl*^ and *Kat*8^*fl/fl*^-*LysMCre* mice were polarized into M2 macrophages with recombinant murine IL-4 or M1 macrophages under LPS and IFN-γ. KAT8 deficiency significantly impaired M2 polarization, as evidenced by a reduced proportion of CD206^+^ macrophages (Figures 3J, 3K, and S3E), decreased ARG1 protein levels (Figure 3L), and reduced expression of M2 hallmark genes (*Arg1*, *Fizz1*, and *Ym1*) (Figures 3M–3O). However, KAT8 deficiency exhibited no significant effects on M1 macrophages polarization, without any apparent influence on *Nos2*, *Tnf-α*, *Il-1β,* and *Il-6* expression (Figures S3F–S3I). Collectively, these findings indicate that KAT8 promotes M2 macrophage polarization in asthma.

### KAT8 interacts with STAT3 and targets it for acetylation

**Figure 4:**
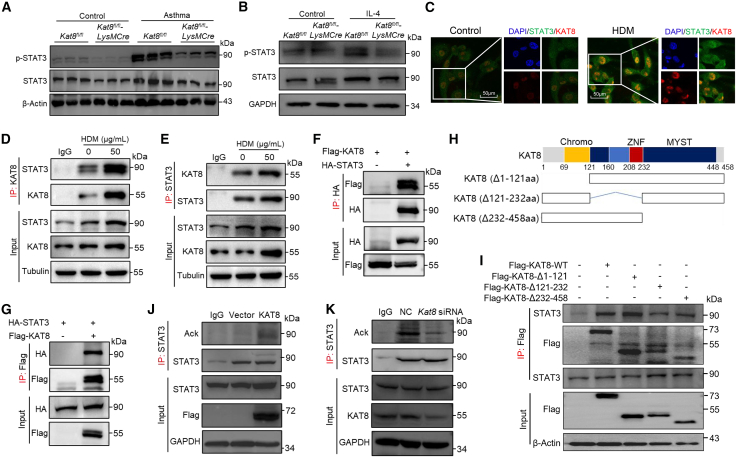
KAT8 interacts with STAT3 and targets it for acetylation (A and B) Phosphorylated and total STAT3 protein levels in lung tissues (A) and IL-4-stimulated BMDMs (B) from *Kat8*^*fl/fl*^ and *Kat8*^*fl/fl*^*-LysMCre* mice (*n* = 3). (C) IF staining shows co-localization of STAT3 (green) and KAT8 (red) in HDM-stimulated BMDMs. Scale bars, 50 μm. (D–G) CoIP analysis of endogenous and exogenous interaction between KAT8 and STAT3 in MH-S cells (D, E) and HEK293T cells (F, G). (H) Mapping of functional domains using truncated KAT8 constructs. (I) CoIP analysis of the interaction between KAT8 truncates and STAT3 in HEK293T cells. (J and K) IP analysis of STAT3 acetylation in HEK293T cells with KAT8 overexpression (J) or *Kat8* siRNA-mediated knockdown (K). Data represent two or three independent experiments.

Previous studies have established STAT3 as a critical mediator of M2 macrophage polarization and a key contributor to asthma pathogenesis.^20^ Therefore, we hypothesize that KAT8 modulates M2 macrophage polarization by regulating STAT3 signaling. Our results demonstrated that KAT8 deficiency significantly downregulated phosphorylated STAT3 (p-STAT3) and total STAT3 protein levels in the lung of asthmatic mice (Figure 4A). Consistent with these *in vivo* findings, KAT8-deficient BMDMs exhibited reduced p-STAT3 and total STAT3 protein levels compared to wild-type (WT) controls after allergen stimulation (Figure 4B). Furthermore, IF staining revealed nuclear co-localization of KAT8 and STAT3 in BMDMs upon HDM stimulation (Figure 4C). Co-immunoprecipitation (Co-IP) assays confirmed an endogenous interaction between KAT8 and STAT3 in HDM-stimulated MH-S cells (Figures 4D and 4E). To validate their association, we transfected HEK293T cells with KAT8 and STAT3 plasmids and revealed a direct exogenous interaction between these two proteins (Figures 4F and 4G). Domain mapping further identified the 121–232 amino acid fragment of KAT8 as essential for its binding to STAT3 (Figures 4H and 4I). Notably, we found that KAT8 overexpression increased STAT3 acetylation levels, while *Kat8* knockdown reduced STAT3 acetylation (Figures 4J and 4K). Collectively, these findings indicate that KAT8 interacts with and acetylates STAT3 in macrophages, indicating a possible role of KAT8 in governing M2 macrophage polarization via STAT3 signaling.

### KAT8 promotes M2 macrophage polarization and allergic airway inflammation via STAT3 signaling

**Figure 5:**
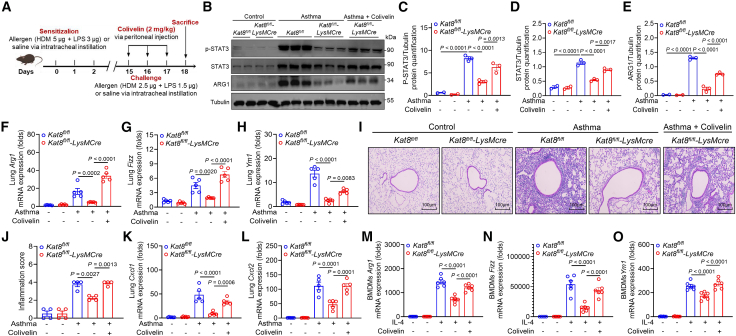
KAT8 promotes M2 macrophage polarization and allergic airway inflammation via STAT3 signaling (A) Schematic view of STAT3 agonist Colivelin treatment in asthma murine model. (B–E) Phosphorylated and total STAT3, and ARG1 protein levels in lung tissues (*n* = 3), and quantified in (C-E), respectively. (F–H) qRT-PCR analysis of *Arg1*, *Fizz1*, and *Ym1* expression in lung tissues (*n* = 5). (I and J) HE staining shows airway inflammation in lung tissues (*n* = 4), quantified in (J). Scale bars, 100 μm. (K and L) qRT-PCR analysis of *Cxcl1* and *Cxcl2* expression in lung tissues (*n* = 5). (M–O) qRT-PCR analysis of *Arg1*, *Fizz1*, and *Ym1* expression in IL-4-stimulatd BMDMs with or without Colivelin treatment. Data are presented as mean ± SEM and represent two or three independent experiments (ANOVA tests).

To investigate whether KAT8 drives allergic airway inflammation and M2 macrophage polarization through STAT3 signaling *in vivo*, we intraperitoneally administered the STAT3 agonist, Colivelin, to *Kat8*^*fl/fl*^-*LysMCre* asthmatic mice (Figure 5A). Colivelin treatment restored p-STAT3 and total STAT3 protein levels in lung tissues of *Kat8*^*fl/fl*^-*LysMCre* asthmatic mice (Figures 5B–5D). Notably, Colivelin treatment also rescued ARG1 expression and the expression of M2 hallmark genes (*Arg1*, *Fizz1*, and *Ym1*) in the lungs of *Kat*8^*fl/fl*^-*LysMCre* asthmatic mice (Figures 5B and 5E–5H). Histopathological analyses revealed that Colivelin treatment abolished the protective effects of KAT8 ablation, exacerbating airway inflammation and tissue damage (Figures 5I and 5J). Concomitantly, Colivelin restored the expression of neutrophil-attracting chemokines *Cxcl1* and *Cxcl2* in *Kat*8^*fl/fl*^-*LysMCre* asthmatic mice (Figures 5K and 5L). Consistent with these *in vivo* findings, Colivelin treatment restored the expression of M2 hallmark genes (*Arg1*, *Fizz1*, and *Ym1*) in KAT8-deficient BMDMs stimulated with IL-4 (Figures 5M–5O). Collectively, these findings demonstrate that KAT8 drives M2 macrophage program and exacerbates allergic airway inflammation via STAT3 signaling.

### Chemical inhibition of KAT8 attenuates allergic airway inflammation

**Figure 6:**
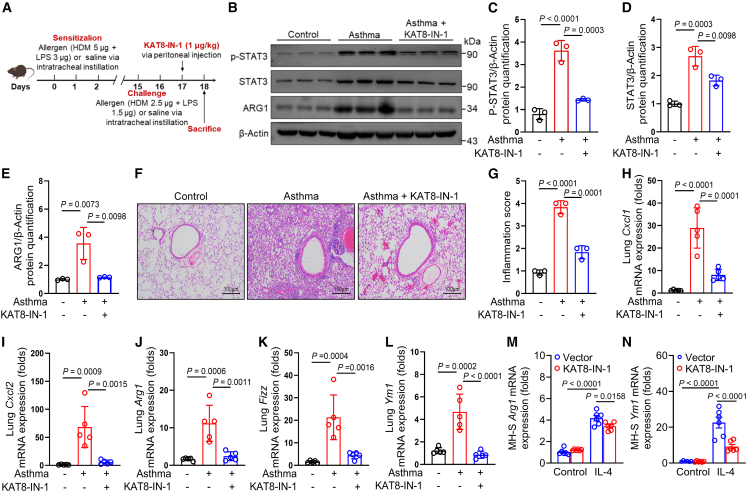
Chemical inhibition of KAT8 attenuates allergic airway inflammation (A) Schematic view of KAT8 inhibitor KAT8-IN-1 treatment in the asthma murine model. (B–E) Phosphorylated and total STAT3, and ARG1 protein levels in lung tissues (*n* = 3), and quantified in (C-E), respectively. (F and G) HE staining shows airway inflammation in lung tissues (*n* = 3), and quantified in (G). Scale bars, 100 μm. (H–L) qRT-PCR analysis of *Cxcl1*, *Cxcl2*, *Arg1*, *Fizz1*, and *Ym1* expression in lung tissues (*n* = 5). (M–N) qRT-PCR analysis of *Arg1* and *Ym1* expression in IL-4-stimulated BMDMs treated with HDM and KAT8-IN-1. Data are presented as mean ± SEM and represent two or three independent experiments (ANOVA tests).

Our findings position KAT8 inhibition as a promising therapeutic strategy for asthma. To validate this, we assessed the efficacy of the KAT8 inhibitor, KAT8-IN-1, in suppressing M2 macrophage polarization and allergic airway inflammation *in vivo* (Figure 6A). KAT8-IN-1 treatment significantly reduced p-STAT3, total STAT3, and ARG1 protein levels in the lungs of asthmatic mice (Figures 6B–6E). Histopathological analyses revealed that KAT8-IN-1 administration markedly attenuated peribronchial inflammatory cell infiltration and tissue damage (Figures 6F and 6G). Furthermore, KAT8-IN-1 treatment downregulated the expression of neutrophil-attracting chemokines (*Cxcl1* and *Cxcl2*) and M2 macrophage markers (*Arg1*, *Fizz1*, and *Ym1*) in lung tissues of asthmatic mice (Figures 6H–6L). In line with these *in vivo* results, KAT8-IN-1treatment also inhibited the expression of M2 hallmark genes (*Arg1* and *Ym1*) in WT BMDMs with IL-4 stimulation (Figures 6M and 6N). Moreover, KAT8-IN-1 treatment suppressed *Cxcl1*, *Cxcl2*, *Arg1*, *Fizz1*, and *Ym1* expression in BMDMs stimulated with HDM (Figures S4A–S4E). These findings suggest that the inhibition of KAT8 alleviates M2 macrophage polarization and allergen-induced airway inflammation in asthma.

## Discussion

Asthma is a heterogeneous chronic lung disease. Dysregulated immune response is a critical driver for asthma pathogenesis. Therefore, understanding the mechanism regulating airway inflammation would help us to develop therapeutic strategies for asthma. Here, we identify KAT8 as a critical epigenetic modulator of allergic airway inflammation. We demonstrate that allergen exposure upregulates KAT8 expression in macrophages, which in turn drives M2 polarization via STAT3 signaling, thereby exacerbating airway inflammation, mucus hypersecretion, and chemokine production in asthma. These findings not only expand the mechanistic understanding of KAT8-mediated STAT3 acetylation-driven asthma pathology but also position KAT8 as a potential therapeutic target for modulating macrophage plasticity in asthma.

Post-translational modifications (PTMs), including acetylation, methylation, and phosphorylation, perform pivotal roles in asthma pathogenesis.^21^ Acetylation, one of the PTMs, has been reported to regulate diverse biological processes, such as gene transcription, DNA repair, protein stability, and cell cycle regulation.^14^^,^^22^ Dysregulated acetylation of histones and non-histone proteins in asthma correlates with disease severity. For example, HAT P300 modulates the asthma-associated gene ORMDL3 via histone acetylation, attenuating airway inflammation and remodeling.^23^ Conversely, histone deacetylase HDAC1 is upregulated in bronchial tissues of severe patients with asthma.^24^ These findings underscore the critical importance of balanced acetylation dynamics in asthma pathogenesis. Our work expands this paradigm by demonstrating that allergen exposure induces dose- and time-dependent expression of KAT8 in macrophages. Genetic ablation of KAT8 in macrophages markedly reduced inflammatory cell infiltration, such as neutrophils, mucus hypersecretion, collagen deposition, and chemokine (CXCL1/CXCL2) expression in the murine asthma model. These findings align with the reports that KAT8 drives IL-33 acetylation in airway epithelial cells, promoting its release and exacerbating inflammation.^18^ Thus, pharmacological inhibition of KAT8 with MG149 or KAT-IN-1 alleviates allergen induced hyperreactivity and airway inflammation.^18^ However, future clinical cohort studies are needed to validate the upregulation of KAT8 specifically in lung macrophages and its correlation with disease severity in patients with asthma.

Macrophage polarization is a central mechanism in asthma pathogenesis. These cells adopt distinct functional phenotypes (e.g., pro-inflammatory M1 or anti-inflammatory M2) in response to microenvironmental cues.^25^^,^^26^ *Robbe* et al. have reported that farm dust extract (FDE) promotes M1 polarization, linked to Th1/Th17 responses, whereas HDM exposure in allergic models skews macrophages toward an M2 phenotype, driving Th2-dominated inflammation.^27^^,^^28^ However, the regulatory mechanisms underlying allergen-induced macrophage polarization remain poorly defined. Here, we identified KAT8 as a critical epigenetic regulator of M2 polarization. KAT8-deficient macrophages exhibited reduced recruitment of M2 macrophages in asthmatic mice and downregulated expression of M2 markers (*Arg1*, *Fizz*, and *Ym1*) under IL-4 polarizing conditions. Moreover, we also found that KAT8 inhibition with KAT8-IN-1 impaired M2 macrophage polarization *in vivo* and *in vitro*. These data suggest that KAT8 is a crucial epigenetic regulator in M2 macrophage polarization in asthma. Emerging evidence highlights the heterogeneity of M2 macrophages, which can be categorized into subsets such as M2a, M2b, M2c, and M2d.^29^^,^^30^ Notably, the M2b subset has garnered increasing attention due to its co-expression of both M1 and M2 surface markers (CD86 and CD206) and its strong pro-inflammatory activity, characterized by the elevated secretion of cytokines and chemokines such as CXCL3, IL-6 and CXCL1,^31^^,^^32^ which can promote tissue inflammation and fibrosis. Although our data demonstrated that KAT8 deficiency inhibited the expression of classic M2 markers, it also reduced the production of CXCL1 and CXCL2 *in vivo* and *in vitro*. Thus, we propose that KAT8 may primarily influence the polarization and function of the M2b subset in neutrophilic asthma, a hypothesis that warrants further investigation. Moreover, neutrophilic airway inflammation in asthma is closely linked to heightened Th17/IL-17A responses.^33^ Whether KAT8 deficiency in macrophages attenuates this neutrophilic inflammation by altering the Th17 response warrants further investigation. Furthermore, while genetic and pharmacological evidence demonstrate that KAT8 enhances M2 macrophage polarization and allergic airway inflammation, macrophage depletion or adoptive transfer experiments remain needed to clarify the causal link between KAT8-regulated M2 polarization and the observed inflammatory phenotype *in vivo*.

The JAK/STAT3 pathway is a well-established driver of cytokine signaling in allergic inflammation.^34^ IL-4-induced STAT3 phosphorylation and activation is essential for M2 polarization, and its inhibition attenuates airway inflammation.^9^ Our study reveals that KAT8 deficiency reduces phosphorylated STAT3 and total STAT3 protein levels in macrophages. We further demonstrate that KAT8 interacts with STAT3 and targets STAT3 for acetylation. Furthermore, administration of the STAT3 agonist, Colivelin, promoted M2 macrophage polarization and exacerbated allergic airway inflammation *in vivo* and *in vitro*, suggesting that KAT8 potentiates STAT3 signaling to amplify the M2 program. This aligns with our previous work showing that HDAC10 deacetylates STAT3 in macrophages, promoting M2 polarization and allergic airway inflammation.^9^ While we have demonstrated KAT8-mediated STAT3 acetylation is crucial for M2 macrophages polarization, the specific lysine residue targeted and the functional consequence of this modification on STAT3’s transcriptional activity in macrophages remain to be determined and warrant further investigation.

In conclusion, our study elucidates KAT8 as a critical epigenetic player of M2 macrophage polarization and allergic airway inflammation via STAT3 acetylation. By bridging macrophage phenotype determination to allergic airway inflammation, these findings nominate KAT8 as a promising therapeutic target for asthma.

### Limitations of the study

This study has several limitations. First, the HDM/LPS model used in our study primarily recapitulates neutrophilic asthma, and further investigation is needed to determine whether our findings extend to other asthma endotypes, particularly eosinophilic asthma. Second, although the LysM-Cre system targets macrophages, this approach also exhibits leakage into other myeloid cells and alveolar type 2 cells. While experiments with isolated neutrophils did not reveal a significant role in our observed phenotype, potential involvement of other LysM-expressing cell types cannot be completely excluded. Moreover, while the conventional M1/M2 classification framework has provided valuable insights, it is difficult to comprehensively evaluate the complexity and plasticity of macrophages *in vivo*. Future studies using single-cell technologies and expanded marker panels will be essential to fully resolve macrophage heterogeneity and KAT8-dependent responses in allergic inflammation. Finally, while our study included both male and female mice in all experiments with sex-matched controls, the potential influence of biological sex on M2 macrophage polarization and airway inflammation needs to be systematically evaluated.

## Resource availability

### Lead contact

Further information and requests for sources and reagents should be directed to and will be fulfilled by the lead contact, Tianwen Lai (laitianwen2011@163.com).

### Materials availability

This study did not generate new unique reagents.

### Data and code availability

• Data: All data reported in the paper are available from the lead contact upon request.
• Code: This paper does not report original code.
• Other: Any additional information required to reanalyze the data reported in this paper is available from the lead contact upon request.

## Acknowledgments

This research was supported by the 10.13039/501100001809National Natural Science Foundation of China (82170030; 82370038), the 10.13039/501100021171Guangdong Basic and Applied Basic Research Foundation (2024B1515120044, 2023A1515140172), the Dongguan Key Laboratory of Immune Inflammation and Metabolism (20231600400872), the Talent Development Foundation of The First Dongguan Affiliated Hospital of Guangdong Medical University& Foundation of State Key Laboratory of Pathogenesis, the Prevention and Treatment of High Incidence Diseases in Central Asia (SKL-HIDCA-2024-GD2A, SKL-HIDCA-2024-GD2B), the Dongguan Science and Technology of Social Development Program (20231800935682), the Guangdong Provincial Key Laboratory of Autophagy and Major Chronic Non-communicable Diseases (2022B1212030003), and the Special Project for Clinical and Basic Sci&Tech Innovation of Guangdong Medical University (GDMULCJC2024094).

## Author contributions

X. L., H. L., Z. Z., Y. Z., and G. S. conducted experiments and data analysis. J. H., Y. X., R. H., J. Q., Z. F., Z. F., S. L., T. H., Z. X., Y. L., and W. Z. performed data analysis. X. G., J. D., and T. L. analyzed/interpreted results and edited the manuscript. T. L. conceived, designed, and supervised the whole study. X. L. and J. D. wrote the manuscript. All authors read and approved the final manuscript.

## Declaration of interests

The authors declare no competing interests.

## STAR★Methods

### Key resources table

**Table:** 

REAGENT or RESOURCE	SOURCE	IDENTIFIER
**Antibodies**		

Anti-KAT8	Santa Cruz	Cat#sc81765; RRID: AB_2235550
Anti-KAT8	Abcam	Cat#ab200660; RRID: AB_2891127
Anti-F4/80	Proteintech	Cat#29414-1-AP; RRID: AB_2918300
Anti-ARG1	Beyotime	Cat#AF1381; RRID: AB_3740890
Anti-Flag	GenScript	Cat#A00187-100; RRID: AB_1720813
Anti-HA	Abbkine	Cat#ABT2040; RRID: AB_2767968
Anti-β-Actin	Beyotime	Cat#AA128; RRID: AB_2861213
Anti-β-Tubulin	Beyotime	Cat#AF0001; RRID: AB_2922414
Anti-GAPDH	Affinity Biosciences	Cat#AF7021; RRID: AB_2839421
Anti-STAT3	Santa Cruz	Cat#sc8019; RRID: AB_628293
Anti-p-STAT3	Cell Signaling Technology	Cat#9145; RRID: AB_2491009
Anti-ARG1	Affinity Biosciences	Cat#DF6657; RRID: AB_2838619

**Biological samples**		

Mouse Lung	This paper	N/A

**Chemicals, peptides, and recombinant proteins**		

Colivelin	SparkJade	Cat#SJ-BP0032
HDM	GREER	Cat#XPB82D3A25
LPS	SIGMA	Cat#L2880-25MG
KAT8-IN-1	MedChemExpress	Cat#HY-W015239
DAPI	Beyotime	Cat#C1005
Protein A+G Agarose	Beyotime	Cat#P2055-50ml
IL-4	Beyotime	Cat#P5916
Recombinant mouse M-CSF	Novoprotein	Cat#CB34
IFN-γ	MedChemExpress	Cat#HY-P78295
RNAi-Mate	GenePharma	Cat#G04001
CD45 (PE/Cyamine7)	Biolegend	Cat#157603; RRID: AB_2876536
F4/80 (APC)	Biolegend	Cat#123115; RRID: AB_893493
CD206 (Percp-Cy5.5)	Biolegend	Cat#141715; RRID: AB_2561991
PerCP/Cyanine5.5 Rat IgG2a, κ Isotype Ctrl Antibody	Biolegend	Cat#400531; RRID: AB_2864286
Fixable Viability Dye eFluor™ 780	Invitrogen	Cat#65-0865-14

**Critical commercial assays**		

Mouse GROα/CXCL1 ELISA Kit	Elabscience	Cat#E-EL-M0018c
Mouse GROβ/CXCL2 ELISA Kit	Elabscience	Cat#E-EL-M0019c
TB Green® Premix Ex Taq™	Takara	Cat#RR420A
Prime Script™ RT reagent Kit	Takara	Cat#RR047A

**Experimental models: Cell lines**		

HEK293T	ATCC	N/A
MH-S	ATCC	N/A

**Experimental models: Organisms/strains**		

Mouse: C57BL/6J (Wild type)	GemPharmatech	N/A
Mouse: *LysMCre*	Dr. G. Feng (The University of California)	N/A
Mouse: *Kat8*^*fl/fl*^	This paper	N/A

**Oligonucleotides**		

siRNA *Kat8* sequence: Forward:GCCGA GAGGAAUUCUAUGUTT Reverse:ACAUAGAAUUCCUCUCGGCTT	Genema	N/A
Primer: Mouse-*Kat8* Forward:TCACTCGAAACCHAAAGCGAA Reverse:AGTTCCCAATGTGGATCTTGTCR	This paper	N/A
Primer: Mouse-*Cxcl1* Forward:CTGGGATTCACCTCAAGAACATC Reverse:CAGGGTCAAGGCAAGCCTC	This paper	N/A
Primer: Mouse-*Cxcl2* Forward:TGTCCCTCAACGGAAGAACC Reverse:CTCAGACAGCGAGGCACATC	This paper	N/A
Primer: Mouse-*β-actin* Forward:AGAGGGAAATCGTGCGTGAC Reverse:CAATAGTGATGACCTGGCCGT	This paper	N/A
Primer: Mouse-*Arg1* Forward:CTGACCTATGTGTCATTTGG Reverse:CATCTGGGAACTTTCCTTTC	This paper	N/A
Primer: Mouse-*Ym1* Forward:GGGCATACCTTTATCCTGAG Reverse:CCACTGAAGTCATCCATGTC	This paper	N/A
Primer: Mouse-*Fizz1* Forward:TCCAGTGAATACTGATGAGA Reverse:CCACTCTGGATCTCCCAAGA	This paper	N/A
Primer: Mouse-*Tnf-α* Forward:TAGCCCACGTCGTAGCAAAC Reverse:ACCCTGAGCCATAATCCCCT	This paper	N/A
Primer: Mouse-*Il-1β* Forward:GCAACTGTTCCTGAACTCAACT Reverse:ATCTTTTGGGGTCCGTCAACT	This paper	N/A
Primer: Mouse-*Il-6* Forward:TAGTCCTTCCTACCCCAATTTCC Reverse:TTGGTCCTTAGCCACTCCTTC	This paper	N/A
Primer: Mouse-*Nos2* Forward:CTCTACAACATCCTGGAGCAAGTG Reverse:ACTATGGAGCACAGCCACATTGA	This paper	N/A

**Recombinant DNA**		

pcDNA3.1-Flag	Yubo Biotechnology, China	
Flag-KAT8 WT	Yubo Biotechnology, China	
Flag-KAT8 Δ1-121	Yubo Biotechnology, China	
Flag-KAT8 Δ121-232	Yubo Biotechnology, China	
Flag-KAT8 Δ232-458	Yubo Biotechnology, China	
HA-STAT3	Yubo Biotechnology, China	

**Software and algorithms**		

GraphPad Prism 8.0	https://www.graphpad.com	N/A
Image J	https://imagej.net/Welcome	N/A
Real-Time PCR Systems	Applied Biosystem	N/A

### Experimental model and Study participant details

#### Animals

The *LysMCre* mice (C57BL/6J background) were provided by Dr. G. Feng (University of California, San Diego, USA). The *Kat8*^*fl/fl*^ mice (C57BL/6J background) was generated using CRISPR-Cas9 technology (GemPharmatech Co., Ltd., Nanjing). To obtain myeloid-specific *Kat8* knockout mice, *Kat8*^*fl/fl*^ animals were crossed with *LysMCre* transgenic mice. Wild-type C57BL/6J controls were acquired from GemPharmatech (Nanjing). All animals were age-matched and sex-matched, and then randomized into different groups. The age for all strains of mice are 6-8 weeks. All mice were maintained in specific pathogen-free animal facilities at the Animal Care Facility of Guangdong Medical University. The housing conditions for the mice like dark/light cycle is 12 h, the ambient temperature is 20-25°C degrees centigrade and the relative humidity is 40-70%. This study was conducted in compliance with institutional animal care guidelines, with all experimental protocols approved by the Animal Ethics Committee of Guangdong Medical University.

#### Allergic asthma mouse model

An experimental asthma model was induced in mice using our previously published methodology.^19^ Mice (6-8 weeks) underwent HDM/LPS sensitization (5/3 μg, days 0-2) followed by challenge (2.5/1.5 μg, days 15-17), both delivered intratracheally in 50 μL saline. Saline-treated controls were compared with two intervention groups receiving either STAT3 agonist or KAT8 inhibitor (intraperitoneal, -2 h relative to challenge). All animals were sacrificed 24 h post-challenge under anesthesia.

#### Cell culture

Standard culture conditions were applied for both cell lines: DMEM medium with 10% fetal bovine serum and 1% antibiotics (penicillin/streptomycin) was used for MH-S alveolar macrophages and HEK293T cells.^9^ BMDMs were isolated from C57BL/6J mice or *kat8*^*fl/fl*^-*LysMCre* mice and cultured in differentiation medium (DMEM with 10% FBS, 1% antibiotics, and 10 ng/mL M-CSF) for 7 days. BMDMs polarizing toward the M2 phenotype was accomplished by 24-hour IL-4 stimulation (10 ng/mL). BMDMs were polarized toward M1 phenotype with LPS (100 ng/mL) plus IFN-γ (20 ng/mL) for 24 hours. Neutrophils were isolated from C57BL/6J mice and maintained in RPMI-1640 supplemented with 10% FBS and 1% antibiotics (penicillin/streptomycin).

### Method details

#### Neutrophils isolation

Neutrophils were isolated from mouse bone marrow following a standardized protocol. Briefly, femurs and tibias were aseptically dissected, and both ends of the bones were trimmed to expose the marrow cavity. Bone marrow cells were flushed out with pre-cooled culture medium into a culture dish and gently dispersed into a single-cell suspension by repeated pipetting. After erythrocyte lysis treatment, the cells were purified using a discontinuous Percoll density gradient centrifugation method. Neutrophils were collected from the high-/low-density interface, washed with PBS to remove residual separation medium, and finally resuspended in a RPMI-1640 supplemented with 10% FBS for subsequent experiments.

#### Histological and immunohistochemical analysis

Lung tissues and human tracheal mucosa sections were sectioned and stained using hematoxylin & eosin for inflammation assessment, Masson's trichrome for collagen deposition, and periodic acid-Schiff for mucus production. Lung sections for immunohistochemistry or immunofluorescence staining were incubated with primary antibodies including anti-STAT3, anti-KAT8, anti-CD206, anti-iNOS, and anti-F4/80 at a 1:200 dilution, followed by secondary antibodies conjugated with Alexa Fluor 594 or 488.Then, the lung sections were incubated with DAPI for 15 minutes. Images were visualized and captured using an Olympus laser scanning confocal microscope. Two independent blinded researchers analyzed ≥10 bronchioles per slide.

#### Western blot

Protein samples (RIPA-extracted, BCA-quantified) were resolved by SDS-PAGE, transferred to PVDF, and immunoblotted with primary antibodies (4°C, overnight). HRP-secondary antibodies (1:5000) enabled chemiluminescent detection.

#### Co-immunoprecipitation (Co-IP)

Protein extracts (1 mg) were immunoprecipitated using target-specific antibodies (1 μg, 4°C, overnight) coupled to Protein G beads (2 h incubation). After completing five washing cycles with lysis buffer, the immunoprecipitated complexes were resolved by SDS-PAGE and detected through immunoblotting analysis.

#### ELISA

Quantification of CXCL1 and CXCL2 in supernatant of BMDMs and lung homogenates was performed using Elabscience ELISA kits, strictly adhering to the manufacturer's technical specifications.

#### Flow cytometry

Following anesthesia, mouse lung tissues were perfused with PBS until visibly blanched, then minced and digested with 1 mg/mL collagenase type I at 37°C for 1 hour. The digested tissues were mechanically dissociated through a 70-μm cell strainer to obtain a single-cell suspension. After centrifugation and washing, red blood cells were lysed using 2 mL of lysis buffer on ice for 5 minutes. The lysis was terminated with 10 mL PBS, and the purified cells were resuspended at a concentration of 1×10^7^ cells/mL. After counting, 1×10^6^ cells were resuspended and incubated in the dark with fixable viability dye (eFluor™ 780) for 10 min at room temperature, followed by washing with PBS. Then, the cells were incubated with CD45-PE/Cy7 and F4/80-APC monoclonal antibodies on ice for 30 minutes in the dark. After washed with PBS containing 1% FBS, the cells were then fixed with 500 μL fixation buffer at room temperature for 20 minutes in the dark. Subsequently, cells were permeabilized and washed with 1× Intracellular Staining Perm Wash Buffer, and then stained with CD206-PerCP/Cy5.5 monoclonal antibodies or its isotype control antibody on ice for 30 minutes. After washed twice, the cell pellets were resuspended in 300 μL of PBS containing 1% FBS for flow cytometry analysis.

Following 12-hour IL-4 stimulation (10 ng/mL), BMDMs were harvested for surface staining with CD45-PE/Cy7 and F4/80-APC monoclonal antibodies, and then followed by intracellular staining with CD206-PerCP/Cy5.5 monoclonal antibodies. After washing, flow cytometric analysis was performed on a FACSCanto II instrument, with data analysis conducted in FlowJo v10.

#### RNA isolation and qRT-PCR

Total RNA from macrophages or lung tissues of mice was extracted using TRIzol (Takara) and then reversely transcribed into first strand cDNA using the RT Reagent Kit (Takara, #RR047A) according to the manufacture’s instruction. qRT-PCR was performed using TB Green Premix Ex Taq II (Takara, #RR420A). The relative expression of individual genes was normalized to *β-actin*. The primers used in this study are listed in key resources table.

#### Bronchoalveolar lavage fluid

Bronchoalveolar lavage fluid (BALF) was performed in anesthetized mice following tracheal exposure and intubation, including three repeated instillations and retrievals of 0.4 mL PBS. The collected BAL fluid was centrifuged at 400 × g for 10 min at 4°C, and the cell pellet was resuspended in 200 μL PBS. Cytospin slides were prepared from 50 μL of the cell suspension by centrifugation at 2500 rpm for 5 min. After air-drying, slides were stained using the Wright-Giemsa method, including incubation with 100 μL of Solution A for 2 min, followed by 100 μL of Solution B for an additional 2 min, with gentle mixing via repeated pipetting. Then, the stained slides were gently rinsed with tap water, air-dried, and examined under a microscope for differential cell counting, including eosinophils, neutrophils, macrophages, and lymphocytes. All counts were performed by two independent blinded researchers.

#### siRNA and plasmids transfection

For siRNA transfection in MH-S cells, RNAi-Mate transfection reagent was used when cells reached 60-80% confluency in 12-well plates, including incubation of siRNA at a final concentration of 25 nM (synthesized by Genema, *Kat8* sequence: 5ʹ-GCCGAGAGGAAUUCUAUGUTT-3ʹ, 5ʹ-ACAUAGAAUUCCUCUCGGCTT-3ʹ) with the transfection reagent diluted separately in OPTI-MEM, followed by mixing and incubation at room temperature for 10-15 minutes. The mixture was then added to serum-containing medium, replaced after 4-6 hours, and transfected for 48 hours in total.

For plasmid transfection in HEK293T cells, RNAi-Mate reagent was applied at approximately 60% confluency in 6-well plates, including direct mixing of 3 μg plasmid (synthesized by Genechem Co) with 3 μL transfection reagent, followed by room temperature incubation for 10-15 minutes and addition to serum-containing medium. The medium was replaced after 24 hours for subsequent treatments.

### Quantification and statistical analysis

Quantitative data are shown as means ± standard error measurements (SEM). The two groups of data were analyzed using unpaired two-tailed Student's t test. Multiple group comparisons were analyzed using one-way ANOVA followed by Tukey's multiple comparisons test. Categorical variables were assessed by chi-square test. All statistical computations were executed in GraphPad Prism 9.0 software, and statistical significance was defined as *P* < 0.05.

## References

[1] Reddel H.K., Bacharier L.B., Bateman E.D., Brightling C.E., Brusselle G.G., Buhl R., Cruz A.A., Duijts L., Drazen J.M., FitzGerald J.M. (2022). Global Initiative for Asthma Strategy 2021. Executive Summary and Rationale for Key Changes. Arch. Bronconeumol..

[2] Porsbjerg C., Melén E., Lehtimäki L., Shaw D. (2023). Asthma. Lancet (London, England).

[3] Papi A., Brightling C., Pedersen S.E., Reddel H.K. (2018). Asthma. Lancet (London, England).

[4] Britt R.D., Ruwanpathirana A., Ford M.L., Lewis B.W. (2023). Macrophages Orchestrate Airway Inflammation, Remodeling, and Resolution in Asthma. Int. J. Mol. Sci..

[5] Locati M., Curtale G., Mantovani A. (2020). Diversity, Mechanisms, and Significance of Macrophage Plasticity. Annu. Rev. Pathol..

[6] Li M., Yang Y., Xiong L., Jiang P., Wang J., Li C. (2023). Metabolism, metabolites, and macrophages in cancer. J. Hematol. Oncol..

[7] Han X., Liu L., Huang S., Xiao W., Gao Y., Zhou W., Zhang C., Zheng H., Yang L., Xie X. (2023). RNA m(6)A methylation modulates airway inflammation in allergic asthma via PTX3-dependent macrophage homeostasis. Nat. Commun..

[8] Ogulur I., Pat Y., Ardicli O., Barletta E., Cevhertas L., Fernandez-Santamaria R., Huang M., Bel Imam M., Koch J., Ma S. (2021). Advances and highlights in biomarkers of allergic diseases. Allergy.

[9] Zhong Y., Huang T., Huang J., Quan J., Su G., Xiong Z., Lv Y., Li S., Lai X., Xiang Y. (2023). The HDAC10 instructs macrophage M2 program via deacetylation of STAT3 and promotes allergic airway inflammation. Theranostics.

[10] Chung S., Kim J.Y., Song M.A., Park G.Y., Lee Y.G., Karpurapu M., Englert J.A., Ballinger M.N., Pabla N., Chung H.Y., Christman J.W. (2019). FoxO1 is a critical regulator of M2-like macrophage activation in allergic asthma. Allergy.

[11] Lechner A., Henkel F.D.R., Hartung F., Bohnacker S., Alessandrini F., Gubernatorova E.O., Drutskaya M.S., Angioni C., Schreiber Y., Haimerl P. (2022). Macrophages acquire a TNF-dependent inflammatory memory in allergic asthma. J. Allergy Clin. Immunol..

[12] Luo M., Zhao F., Cheng H., Su M., Wang Y. (2024). Macrophage polarization: an important role in inflammatory diseases. Front. Immunol..

[13] van den Bosch T., Kwiatkowski M., Bischoff R., Dekker F.J. (2017). Targeting transcription factor lysine acetylation in inflammatory airway diseases. Epigenomics.

[14] Shvedunova M., Akhtar A. (2022). Modulation of cellular processes by histone and non-histone protein acetylation. Nat. Rev. Mol. Cell Biol..

[15] Chatterjee A., Seyfferth J., Lucci J., Gilsbach R., Preissl S., Böttinger L., Mårtensson C.U., Panhale A., Stehle T., Kretz O. (2016). MOF Acetyl Transferase Regulates Transcription and Respiration in Mitochondria. Cell.

[16] Li L., Ghorbani M., Weisz-Hubshman M., Rousseau J., Thiffault I., Schnur R.E., Breen C., Oegema R., Weiss M.M., Waisfisz Q. (2020). Lysine acetyltransferase 8 is involved in cerebral development and syndromic intellectual disability. J. Clin. Investig..

[17] Dong Z., Zou J., Li J., Pang Y., Liu Y., Deng C., Chen F., Cui H. (2019). MYST1/KAT8 contributes to tumor progression by activating EGFR signaling in glioblastoma cells. Cancer Med..

[18] Liu Y., Du J., Liu X., Wang L., Han Y., Huang C., Liang R., Zheng F., Shi G., Li B. (2021). MG149 inhibits histone acetyltransferase KAT8-mediated IL-33 acetylation to alleviate allergic airway inflammation and airway hyperresponsiveness. Signal Transduct. Target. Ther..

[19] Quan J., Wen X., Su G., Zhong Y., Huang T., Xiong Z., Huang J., Lv Y., Li S., Luo S. (2023). Epithelial SIRT6 governs IL-17A pathogenicity and drives allergic airway inflammation and remodeling. Nat. Commun..

[20] Bi C., Fu Y., Li B. (2020). Brain-derived neurotrophic factor alleviates diabetes mellitus-accelerated atherosclerosis by promoting M2 polarization of macrophages through repressing the STAT3 pathway. Cell. Signal..

[21] Ntontsi P., Photiades A., Zervas E., Xanthou G., Samitas K. (2021). Genetics and Epigenetics in Asthma. Int. J. Mol. Sci..

[22] Narita T., Weinert B.T., Choudhary C. (2019). Functions and mechanisms of non-histone protein acetylation. Nat. Rev. Mol. Cell Biol..

[23] Cheng Q., Shang Y., Huang W., Zhang Q., Li X., Zhou Q. (2020). Corrigendum to “p300 mediates the histone acetylation of ORMDL3 to affect airway inflammation and remodeling in asthma”. Int. Immunopharmacol..

[24] Butler C.A., McQuaid S., Taggart C.C., Weldon S., Carter R., Skibinski G., Warke T.J., Choy D.F., McGarvey L.P., Bradding P. (2012). Glucocorticoid receptor β and histone deacetylase 1 and 2 expression in the airways of severe asthma. Thorax.

[25] Liu Y., Liu P., Duan S., Lin J., Qi W., Yu Z., Gao X., Sun X., Liu J., Lin J. (2025). CTCF enhances pancreatic cancer progression via FLG-AS1-dependent epigenetic regulation and macrophage polarization. Cell Death Differ..

[26] Dousdampanis P., Aggeletopoulou I., Mouzaki A. (2024). The role of M1/M2 macrophage polarization in the pathogenesis of obesity-related kidney disease and related pathologies. Front. Immunol..

[27] Saradna A., Do D.C., Kumar S., Fu Q.L., Gao P. (2018). Macrophage polarization and allergic asthma. Transl. Res..

[28] Robbe P., Draijer C., Borg T.R., Luinge M., Timens W., Wouters I.M., Melgert B.N., Hylkema M.N. (2015). Distinct macrophage phenotypes in allergic and nonallergic lung inflammation. Am. J. Physiol. Lung Cell. Mol. Physiol..

[29] Tang P.M.K., Nikolic-Paterson D.J., Lan H.Y. (2019). Macrophages: versatile players in renal inflammation and fibrosis. Nat. Rev. Nephrol..

[30] Rőszer T. (2015). Understanding the Mysterious M2 Macrophage through Activation Markers and Effector Mechanisms. Mediators Inflamm..

[31] Lv H., Huang G., Li H., Liang H., Peng H., Wu K., Chen W., Zhang D., Ma K., Du Y. (2026). Reprogramming M2b Macrophages via GPX1 Activation by Selenium Nanoparticles Attenuates Lupus Nephritis. Adv. Sci..

[32] Qian J., Tao Q., Shen Y., Wang L., Wang M., Wang N., Liang Q., Lu J., Huang Y., Liao W. (2025). Periodontitis salivary microbiota exacerbates colitis by CXCL3 derived from gut microbiota-induced macrophages. Microbiome.

[33] Han S., Kim B., Hyeon D.Y., Jeong D., Ryu J., Nam J.S., Choi Y.H., Kim B.R., Park S.C., Chung Y.W. (2024). Distinctive CD39(+)CD9(+) lung interstitial macrophages suppress IL-23/Th17-mediated neutrophilic asthma by inhibiting NETosis. Nat. Commun..

[34] Guo H., Zhao Y., Zhang Z., Xu Y., Chen Y., Lei T., Zhao Y. (2025). The Presence and Pathogenic Roles of M(IL-33 + IL-2) Macrophages in Allergic Airway Inflammation. Allergy.

