# Supplementary material for: KAT8 drives M2 macrophage polarization to exacerbate allergic airway inflammation
Source: iScience. 2026 Mar 12;29(4):115348. doi: 10.1016/j.isci.2026.115348 (PMC13053759; doi:10.1016/j.isci.2026.115348)
Supplement: Document S1. Figures S1–S4 and Data S1 [file mmc1.pdf]

iScience, Volume *29*

Supplemental information

KAT8 drives M2 macrophage polarization to exacerbate allergic airway inflammation

Xianwen Lai, Han Li, Zhao Zhao, Yu Zhong, Guomei Su, Jiewen Huang, Yuanyuan Xiang, Ruina Huang, Jingyun Quan, Zhihang Feng, Zhenfu Fang, Shihai Li, Tong Huang, Zhiling Xiong, Yuting Lei, Wenchao Zhang, Jielin Duan, Xiao Gao, and Tianwen Lai

Supplemental Text and Figures

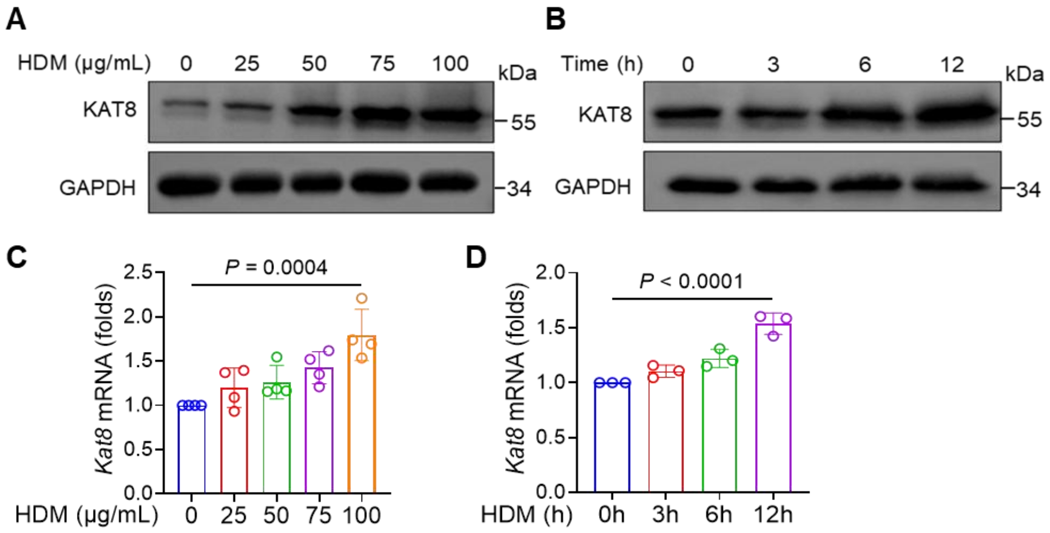

Figure. S1 HDM exposure induces KAT8 expression in MH-S cells, related to Fig 1

(A-D) WB (A, B) and qRT-PCR (C, D) analysis of KAT8 expression in HDM-stimulated MH-S cells.

Data are presented as mean ± SEM and represent two or three independent experiments (ANOVA tests).

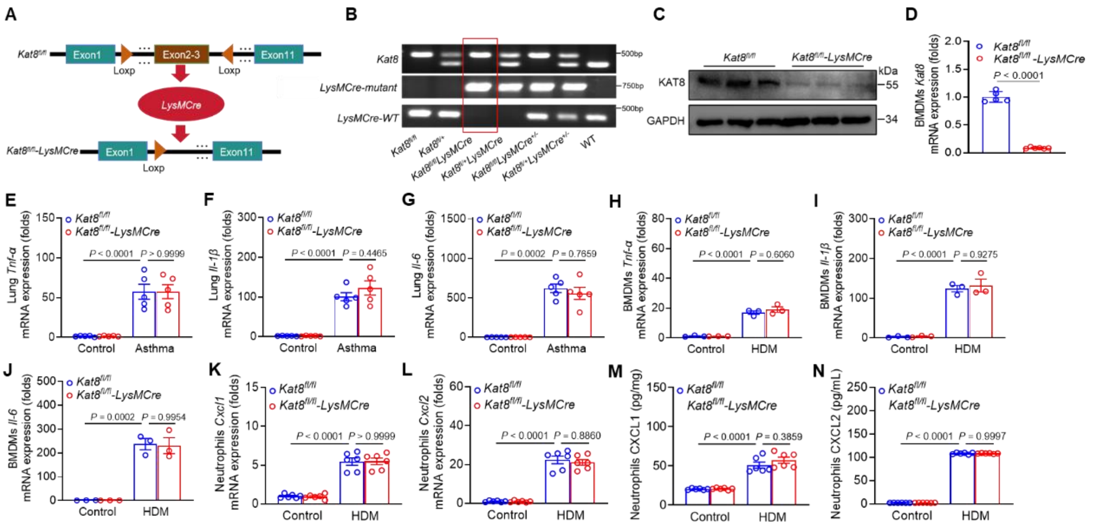

Figure. S2 KAT8 deficiency in macrophages exhibit no significant effects on pro-inflammatory cytokines expression, related to Fig 2.

(A) Cre/loxP strategy for myeloid-specific *Kat8* deletion.

(B) Genotyping PCR confirming *Kat8$^{fl/fl}$-LysMCre* mice.

(C, D) KAT8 protein (C) and mRNA (D) levels in BMDMs from *Kat8$^{fl/fl}$* and *Kat8$^{fl/fl}$-LysMCre* mice.

(E-G) The expression of *Tnf-α*, *Il-1β*, and *Il-6* in lung tissues from *Kat8$^{fl/fl}$* and *Kat8$^{fl/fl}$-LysMCre* asthmatic mice (n=5).

(H-J) The expression of *Tnf-α*, *Il-1β*, and *Il-6* in BMDMs from *Kat8$^{fl/fl}$* and *Kat8$^{fl/fl}$-LysMCre* mice.

(K-N) CXCL1 and CXCL2 expression and production in HDM-stimulated neutrophils from *Kat8$^{fl/fl}$* and *Kat8$^{fl/fl}$-LysMCre* mice, analyzed by qRT-PCR (K, L) and ELISA (M, N).

Data are presented as mean ± SEM and represent two or three independent experiments (Student's t tests and ANOVA tests).

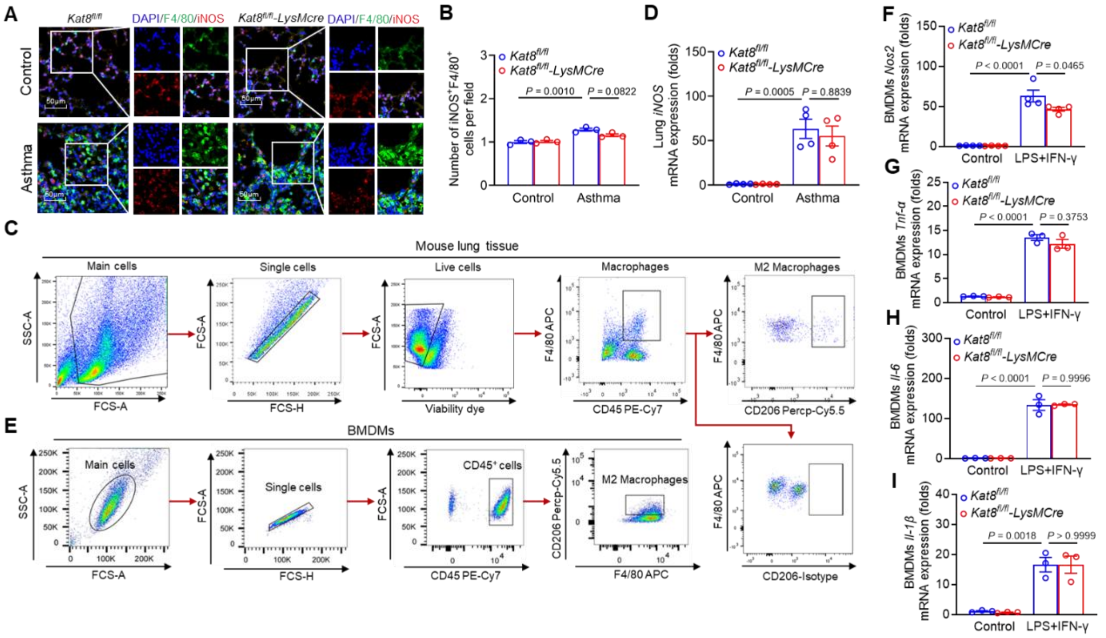

Figure. S3 KAT8 deficiency in macrophages exhibit no effect on M1 macrophages polarization, related to Fig 3

(A, B) IF staining showing M1 macrophages (iNOS$^+$F4/80$^+$) in the lung of *Kat8$^{fl/fl}$* and *Kat8$^{fl/fl}$-LysMCre* asthmatic mice (n=3), and quantified in (B). Scale bar, 50 µm.

(C) Flow cytometry gating strategy for M2 macrophages in lung tissues (n=4).

(D) The expression of *Nos2* in lung tissues from *Kat8$^{fl/fl}$* and *Kat8$^{fl/fl}$-LysMCre* asthmatic mice (n=4).

(E) Flow cytometry gating strategy for M2 macrophages in IL-4-stimulated BMDMs.

(F-I) The expression of *Nos2*, *Tnf-α*, *Il-1β*, and *Il-6* in BMDMs from *Kat8$^{fl/fl}$* and *Kat8$^{fl/fl}$-LysMCre* mice.

Data are presented as mean ± SEM and represent two or three independent experiments (ANOVA tests).

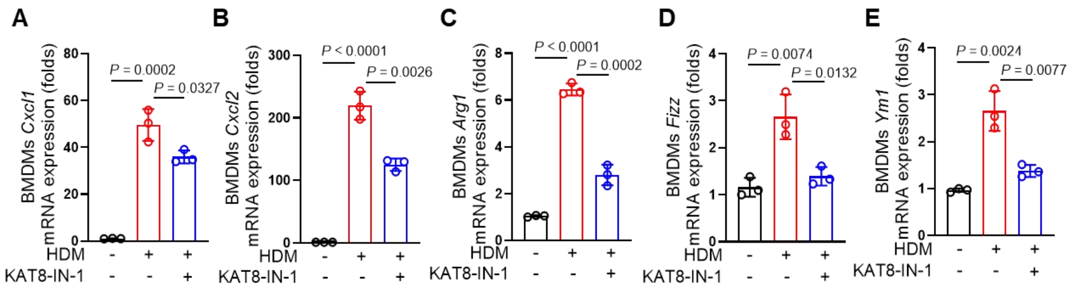

Figure. S4 KAT8-IN-1 treatment impairs M2 macrophages polarization

(A-E) qRT-PCR analysis of *Cxcl1*, *Cxcl2*, *Arg1*, *Fizz1*, and *Ym1* expression in BMDMs treated with HDM and KAT8-IN-1.

Data are presented as mean ± SEM and represent two or three independent experiments (ANOVA tests).

Data S1. Original image for WB

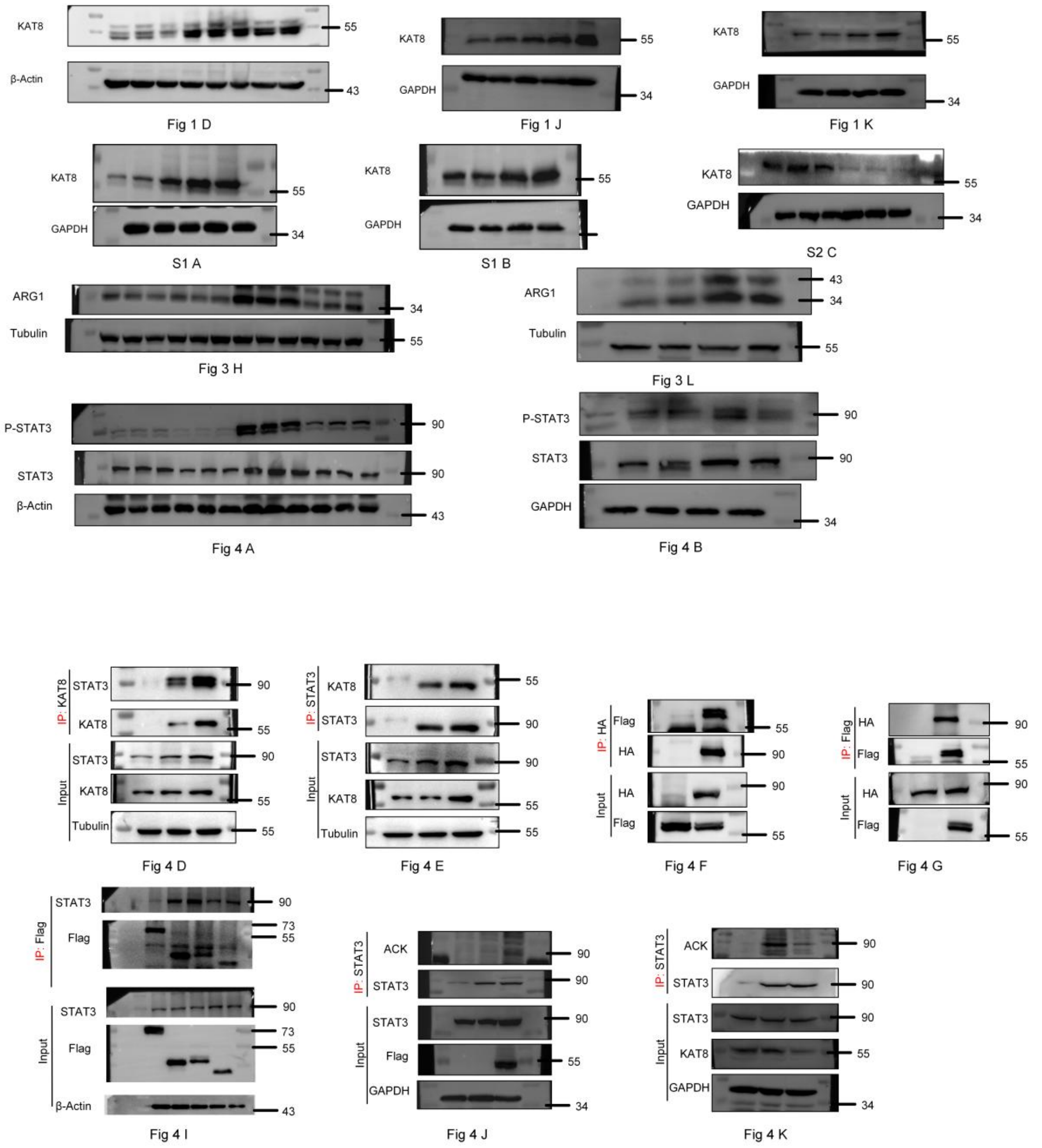

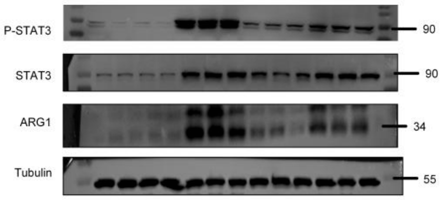
P-STAT3
90
STAT3
90
ARG1
34
Tubulin
55
Fig 5 B

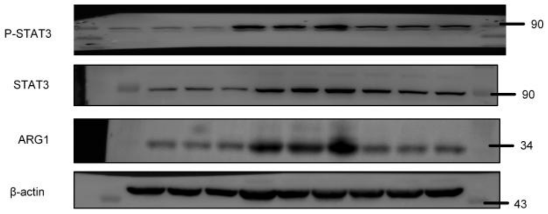
P-STAT3
90
STAT3
90
ARG1
34
β-actin
43
Fig 6 B
